# Visual information without thermal energy may induce thermoregulatory-like cardiovascular responses

**DOI:** 10.1186/1880-6805-32-26

**Published:** 2013-12-28

**Authors:** Jun'ya Takakura, Takayuki Nishimura, Shigeki Watanuki

**Affiliations:** 1 Graduate School of Integrated Frontier Science, Kyushu University, 4-9-1, Shiobaru, Minami-Ku, Fukuoka, Japan; 2 Department of Public Health, Nagasaki University Graduate School of Biomedical Sciences, 1-12-4, Sakamoto, Nagasaki, Japan; 3 Faculty of Design, Kyushu University, 4-9-1, Shiobaru, Minami-Ku, Fukuoka, Japan

**Keywords:** Video, Visual information, Impressions, Cardiovascular responses, Physiological thermoregulatory systems, Virtual reality

## Abstract

**Background:**

Human core body temperature is kept quasi-constant regardless of varying thermal environments. It is well known that physiological thermoregulatory systems are under the control of central and peripheral sensory organs that are sensitive to thermal energy. If these systems wrongly respond to non-thermal stimuli, it may disturb human homeostasis.

**Methods:**

Fifteen participants viewed video images evoking hot or cold impressions in a thermally constant environment. Cardiovascular indices were recorded during the experiments. Correlations between the ‘hot-cold’ impression scores and cardiovascular indices were calculated.

**Results:**

The changes of heart rate, cardiac output, and total peripheral resistance were significantly correlated with the ‘hot-cold’ impression scores, and the tendencies were similar to those in actual thermal environments corresponding to the impressions.

**Conclusions:**

The present results suggest that visual information without any thermal energy can affect physiological thermoregulatory systems at least superficially. To avoid such ‘virtual’ environments disturbing human homeostasis, further study and more attention are needed.

## Background

Maintaining a quasi-constant core body temperature is essential for sustaining human life. This physiological temperature regulation system can be roughly divided into two parts comprising sensors and effectors. Sensors have sensitivity to thermal energy (temperature), and are distributed throughout the central and peripheral body areas. Sensory thermal information is integrated in the preoptic area of the hypothalamus, which is thought to be the central component of thermoregulation [1]. According to this sensory information, the hypothalamus then drives effectors. For example, in a situation where the body temperature should be lowered, vasodilation and increased cardiac output occur to facilitate heat radiation. In contrast, in a situation where body temperature should be elevated, vasocontraction and decreased cardiac output occur to hinder heat radiation [2-4].

This human thermoregulatory system is often compared to manmade systems [5], such as an air conditioner, which also has sensors and effectors that work to maintain a constant air temperature. These systems work because engineers select the appropriate sensors (for example, a thermistor, which senses temperature) to detect thermal energy. If inappropriate sensors (for example, a phototransistor, which senses visible light) are selected and embedded in the system, temperature regulation would be disturbed [6], as visible light has almost no thermal energy.

Unlike manmade systems, however, the human thermoregulatory system developed over the course of evolution rather than being designed by rational engineers [7]. Thus, inappropriate sensors may have been incorporated into the human thermoregulatory system. Although a wholly inadequate design would not have been selected in the course of evolution, a design that is imperfect but did not have destructive consequences in the context of evolution may have been able to persist [8]. Assuming that thermal stimuli are always accompanied by non-thermal stimuli, then sensitivity to non-thermal stimuli is synonymous with sensitivity to thermal stimuli, because it can act as actual physiological thermal sensors instead. Thermoregulatory systems that are partly under the control of non-thermal stimuli work well as long as both thermal stimuli and non-thermal stimuli correlate with each other, in which case such a system would not have been maladaptive in the course of human evolution. For example, visual information can act as non-thermal stimuli; for example, presumably, when human ancestors were exposed to a cold environment, visual stimuli such as snow or ice were also apparent.

In today’s environment, media technologies have enabled us to be exposed to visual stimuli corresponding to hot or cold temperatures independent of the actual thermal environment. In other words, thermal stimuli and non-thermal stimuli have begun to dissociate, and if non-thermal stimuli can exert significant effects on thermal regulation, this may have negative consequences. Considering the rapid increase in the use of electronic media [9], the effects of non-thermal stimuli on homeostasis should be considered. The purpose of this study was to examine whether visual information that for human ancestors would have been accompanied by actual thermal stimuli (that is, hot or cold environments) affects human thermoregulatory physiological responses in the absence of any actual thermal stimulus.

## Methods

**Figure 1:**
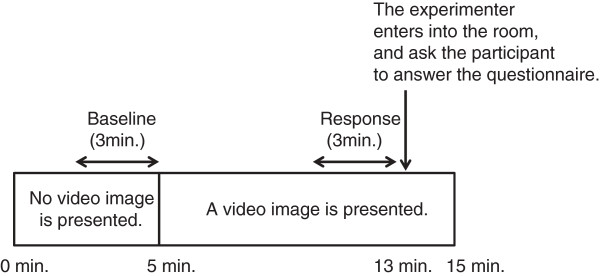
**Experimental protocol.** Participants sit on a chair in front of a display. After the experimenter exits the room, a gray screen is shown for 5 minutes. Then, each video is presented for 10 minutes. After 8 minutes, the experimenter enters the room and participants are told to fill in the questionnaire. The order of the videos is at random. Physiological indices at the last 3 minutes of the gray screen period are used as baseline. The period of 5 minutes to 8 minutes during video image presentation is used to measure response.

**Figure 2:**
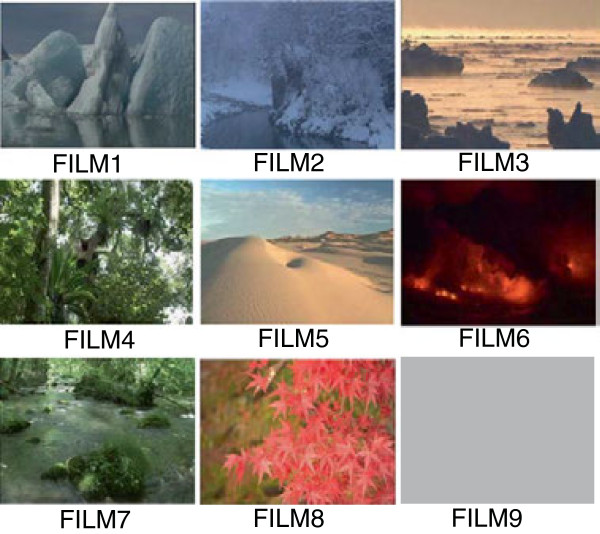
**Examples of videos.** There were nine videos (FILM1 to FILM9) recorded on DVD. The contents of each are: FILM1, icebergs; FILM2, snow; FILM3, drifting ice; FILM4, tropical forest; FILM5, desert; FILM6, erupting volcano; FILM7, stream; FILM8, autumn leaves; and FILM9, gray screen.

Fifteen male graduate and undergraduate students (age, 20 to 24 years old) participated in this study. The experimental protocol consisted of a total of nine sessions. The time course of each session is shown in Figure 1. After the sensors were attached to the participants, they sat on a chair in front of a display (PFM-42B1, Sony, Tokyo, Japan). After 5 minutes of viewing a gray screen, videos were presented for 10 minutes. After viewing the images for 8 minutes, participants rated their subjective impressions of the images and their thermal sensations on a 7-point scale from -3 (very cold) to +3 (very hot). Impressions of the images on a 7-point scale about pleasantness (like-dislike) were also recorded. Here, ‘impressions’ refer to participants’ subjective feelings about the videos and ‘thermal sensations’ refer to their actual physical sensations of temperature. The participants’ drowsiness was checked between the experimental sessions and they were allowed to talk with the experimenter to keep the arousal level. The presented images comprised sceneries of icebergs (FILM1), snow (FILM2), drifting ice (FILM3), a tropical forest (FILM4), a desert (FILM5), an erupting volcano (FILM6), a stream (FILM7), autumn leaves (FILM8), and a gray screen (FILM9). Examples of the images are shown in Figure 2. The nine images were presented in random order to avoid ordering effects. The temperature of the experimental room was set at 28°C and the humidity was set at 50% of relative humidity. The experiment was conducted in autumn in Japan.

Physiological signals were measured continuously throughout the sessions. Electrocardiography (ECG) electrodes were attached to the chest and amplified by an amplifier (AB-621G, Nihon Kohden, Tokyo, Japan). Impedance cardiography was measured using a tape-electrode and an impedance meter (AI-601G, Nihon Kohden), and its time derivation was also calculated (ED-601G, Nihon Kohden). Continuous blood pressure was measured through the finger (Finapress 2300, Ohmeda, Amsterdam, Netherlands). These analog signals were digitalized by an A/D converter at a sampling rate of 1,000 Hz and stored on a personal computer. The experimental procedure was approved by the local ethics committee of the Faculty of Design, Kyushu University (Fukuoka, Japan). All participants provided written informed consent.

Offline analysis was conducted using in-house software developed in our laboratory. Cardiovascular indices were calculated beat-by-beat. Heart rate was derived from the R waves of ECG. Stroke volume was calculated using Kubicek’s formula [10] by derivation from impedance cardiography, which was filtered by an adaptive filter [11]. Systolic and diastolic blood pressures were the upper and lower peaks of the blood pressure waveform. Mean blood pressure was calculated by averaging the waveform. Cardiac output and total peripheral resistance were also calculated based on the above indices. Heart rate variability (HRV) was calculated using the maximum entropy method and the power spectrum was divided into three components: HF (0.15 Hz to 0.40 Hz), LF (0.06 Hz to 0.15 Hz), and VLF (0.02 Hz to 0.06 Hz).

Changes from baseline were calculated for each index: heart rate (ΔHR), stroke volume (ΔSV), cardiac output (ΔCO), systolic blood pressure (ΔSBP), diastolic blood pressure (ΔDBP), mean blood pressure (ΔMBP), total peripheral resistance (ΔTPR), high frequency HRV (ΔHF), low frequency HRV (ΔLF), and very low frequency of HRV (ΔVLF).

To confirm that each video invoked a different impression, we applied the Friedman test to the ‘hot-cold’ impression score. We did not conduct a post-hoc test, because this was not our main purpose.

We used multiple regression analysis to determine the relationship between cardiovascular indices and impression scores among the participants [12]. We used cardiovascular indices as dependent variables, and impression scores and participants as independent variables. Participants were treated as categorical factors using dummy variables with 14 degrees of freedom. The *P* value from the t-test for the regression slope (the common coefficient *a* described as below) of impression scores was used to determine the probability of the analysis. The magnitude of the intra-individual correlation coefficients between the impression score and the cardiovascular indices among the participants was calculated as:

$$|r|=\sqrt{\frac{\text{sum}\;\text{of}\;\text{squares}\;\text{for}\;\text{impression}\;\text{score}}{\text{sum}\;\text{of}\;\text{squares}\;\mathrm{for}\;\text{impression}\;\text{score}+\text{residual}\;\text{sum}\;\text{of}\;\text{square}}}$$

The sign of the correlation coefficients is given by that of the regression coefficients for impression score. Note that the above *r* is not the multiple regression correlation coefficient and the *P* value is not the result of overall multiple regression analysis. Although this method is useful for many purposes, many readers may not be familiar with this method. Thus, we explain this method somewhat minutely.

**Figure 3:**
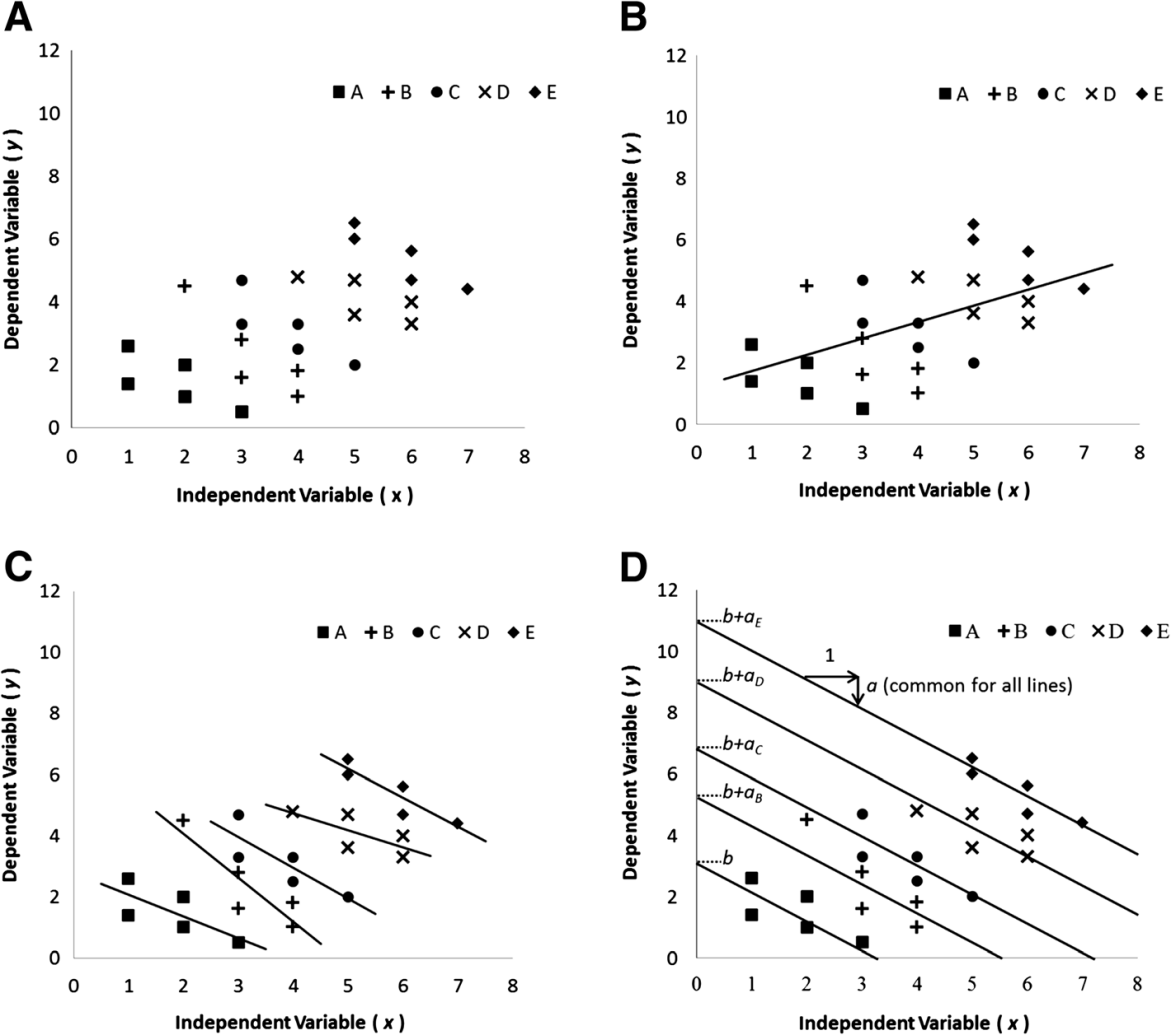
**Explanation of correlation calculations.(A)** Scatter plot of sample data consisting of five participants. **(B)** Falsely detected positive correlation between the independent variable and the dependent variable. **(C)** Intra-individual correlation for each participant. **(D)** Determining intra-individual correlation using multiple regression analysis.

We collected both independent variables (impression scores) and dependent variables (cardiovascular indices) for each participant. The data for each participant comprised several points. If we take the data in Figure 3A, then this sample consists of five participants and each participant has five data points. Applying correlation analysis to these data, there is a positive correlation between independent variables and dependent variables, as shown in Figure 3B. However, looking at the data intra-individually, a negative correlation is seen, as shown in Figure 3C. Multiple regression analysis using dummy variables identified an intra-individual correlation. Here, we establish *y*(*i*, *j*) as the dependent variable and *x*(*i*, *j*) as the independent variable, where *i* denotes the participant (*i* = A, B, C, D, and E) and *j* is the intra-individual data indicator (*j* = 1, 2, … , 5). In addition, we insert dummy independent variables *xd*_k_(*i*, *j*) (*k* = B, C, D, and E), defined as:

$$xd_{k}\left(i,j\right)=\left\{\begin{array}{cc} 1 & i=k\\ 0 & i\neq k \end{array}\right)$$

Now we have one-dimensional dependent variables *y*(*i*, *j*) and five-dimensional independent variables consisting of *x*(*i*, *j*) and *xd*_k_(*i*, *j*). Applying multiple regression analysis to these data, we obtain the following regression formula:

$$\begin{array}{l} \hat{y}\left(i,j\right)=\mathrm{ax}\left(i,j\right)+a_{B}xd_{B}\left(i,j\right)\\ \;+a_{C}xd_{C}\left(i,j\right)+a_{D}xd_{D}\left(i,j\right)+a_{E}xd_{E}\left(i,j\right)+b \end{array}$$

In the above formula, *a* is the common coefficient that explains the intra-individual slopes. Each *a*_*k*_ is biased for each participant. If the common coefficient *a* is non-zero, it means that the dependent variable *y*(*i*, *j*) and the independent variable *x*(*i*, *j*) are correlated intra-individually and that tendency is common among participants (see Additional file 1). Figure 3D is a schematic explanation of the regression formula.

There are possible confounding factors that affect cardiovascular responses other than hot or cold visual information. If the participants feel ‘like’ or ‘dislike’ about the video images, it can also induce cardiovascular responses. In addition, although we set the room air temperature constant, it is unavoidable to fluctuate in some degree, and possibly induce actual thermoregulatory cardiovascular responses. To confirm these possibilities, we conducted the same regression analysis adapting the subjective impression scores of ‘like-dislike’ and room air temperature during each session as independent variables.

These statistical analyses were conducted using statistical software (R 2.12.1, R Development Core Team, Vienna, Austria). All tests were two-sided and *P* <0.05 was regarded as statistically significant. When *P* <0.1 this was also taken into consideration as mentioned in the Discussion.

## Results

**Figure 4:**
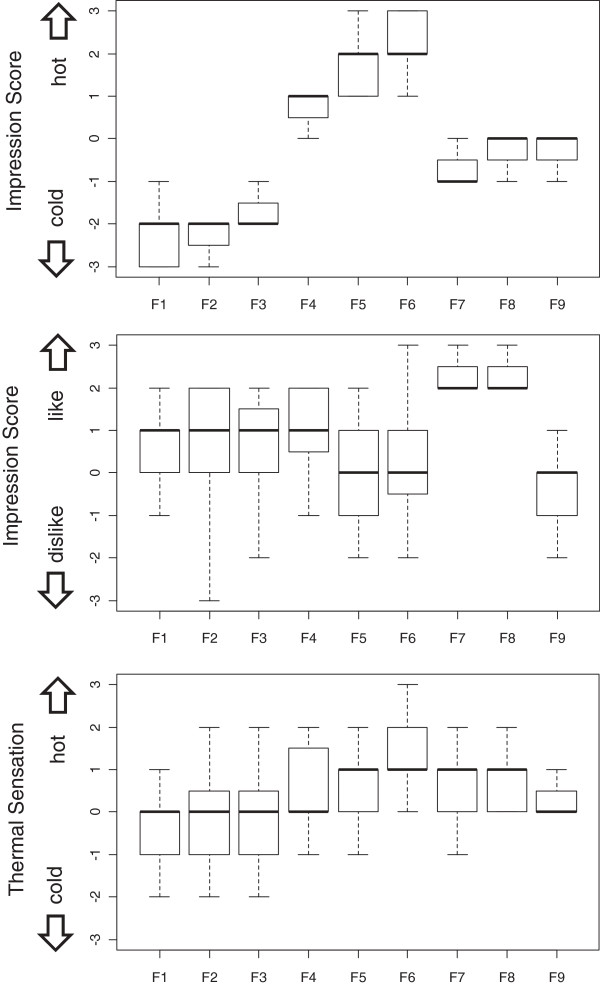
**Box and whisker plot of subjective responses.** The bars in the plot signify the minimum and maximum values excluding extreme outliers. The top and bottom edges of the boxes indicate the 75th percentile and 25th percentile values, respectively. The bold lines on the boxes are the median. In the ‘hot-cold’ impression score, significant differences were found among the values (Friedman test, *P* <0.01).

**Figure 5:**
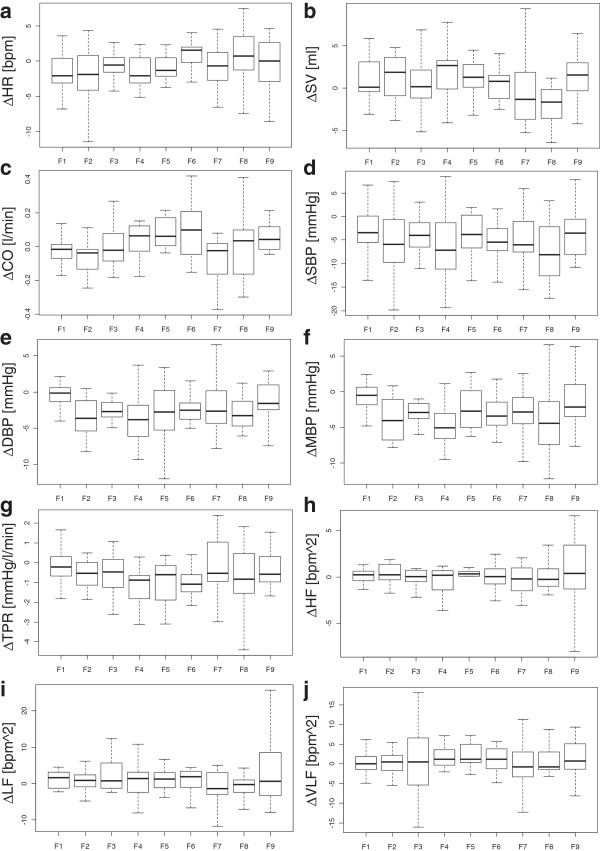
**Box and whisker plot of cardiovascular indices.** Cardiovascular indices for the videos. Changes from baseline: **(A)** ΔHR, heart rate; **(B)** ΔSV, stroke volume; **(C)** ΔCO, cardiac output; **(D)** ΔSBP, systolic blood pressure; **(E)** ΔDBP, diastolic blood pressure; **(F)** ΔMBP, mean blood pressure; **(G)** ΔTPR, total peripheral resistance; **(H)** ΔHF, high frequency HRV; **(I)** ΔLF, low frequency HRV; and **(J)** ΔVLF, very low frequency of HRV. The meanings of the bars, edges, and bold lines are the same as in Figure 4. In ΔCO, significant differences were found among the values (Friedman test, *P* <0.05). HRV, heart rate variability.

Participants’ impression scores for the videos and thermal sensation scores are shown in Figure 4. The Friedman test revealed statistically significant differences among the videos (*P* <0.01), meaning that the images used could successfully invoke different levels of hot or cold impressions. All cardiovascular indices for the videos are shown in Figure 5. In ΔCO, there were significant differences among videos (Friedman test, *P* <0.05). In other cardiovascular indices, the results of Friedman tests did not reach significant level.

**Figure 6:**
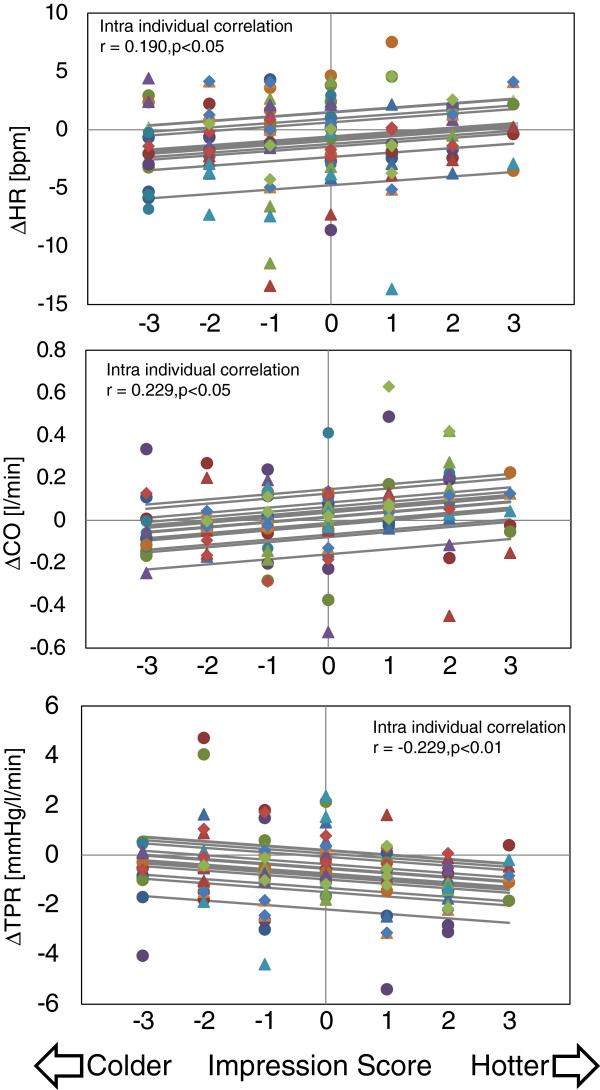
**Scatter plot of ΔHR, ΔCO, and ΔTPR referring to the ‘hot**-**cold’ impression score.** Different colors and shapes in the plots indicate different participants. Regression lines are drawn for all participants, using the common coefficient *a*. Changes from baseline: ΔHR, heart rate; ΔCO, cardiac output; and ΔTPR, total peripheral resistance.

Table 1 shows the overall results of the correlation analysis. There was a significant positive correlation between ‘hot-cold’ impression score and ΔHR (r = 0.190, *P* <0.05), ΔCO (r = 0.229, *P* <0.05), and ΔVLF (r = 0.190, *P* <0.05). Also, a significant negative correlation between the impression score and ΔTPR (r = 0.247, *P* <0.05) was observed. In addition, although it did not reach the level of significance, there was a tendency towards a correlation between the ‘hot-cold’ impression score and ΔDBP (r = 0.157, *P* <0.1) and ΔMBP (r = 0.163, *P* <0.1). There were no significant correlations between ‘hot-cold’ impression scores and ΔSV (r = 0.008, *P* = 0.934), ΔSBP (r = 0.099, *P* = 0.282), ΔLF (r = 0.071, *P* = 0.437), or ΔHF (r = 0.037, *P* = 0.691). Here, a positive correlation means the impression is hotter and the physiological index is greater; a negative correlation means the impression is colder and the physiological index is greater. Not surprisingly, there was also a significant positive correlation between the ‘hot-cold’ impression score and thermal sensation (r = 0.439, *P* <0.01). The raw results of multiple regression analyses are shown in Additional file 2, and the scatter plots of ΔHR, ΔCO, and ΔTPR are shown in Figure 6.

**Table 1:** **Overall results of correlation analysis**: **impression scores of** ‘**hot**-**cold’**

**Indices**	**Intra-individual correlation coefficients(r)**	***P*** **value for null hypothesis is that common coefficient** ***a*** = **0**	**Significance**
ΔHR	+0.190	0.037	^**^
ΔSV	+0.008	0.934	
ΔCO	+0.229	0.012	^**^
ΔSBP	-0.099	0.282	
ΔDBP	-0.157	0.085	^*^
ΔMBP	-0.163	0.074	^*^
ΔTPR	-0.247	0.006	^***^
ΔHF	+0.111	0.224	
ΔLF	+0.071	0.437	
ΔVLF	+0.190	0.036	^**^
Thermal sensation	+0.439	<0.0001	^***^

Correlation coefficients between each index and ‘hot-cold’ impression scores. Positive correlations mean the hotter the impressions, the greater the indices. ^*^P <0.1; ^**^P <0.05; ^***^P <0.01. The *P* values are for the common coefficient *a*, not the results of multiple regression analysis. The raw results of multiple regression analyses are shown in Additional file 2. Changes from baseline: ΔHR, heart rate; ΔSV, stroke volume; ΔCO, cardiac output; ΔSBP, systolic blood pressure; ΔDBP, diastolic blood pressure; ΔMBP, mean blood pressure; ΔTPR, total peripheral resistance; ΔHF, high frequency HRV; ΔLF, low frequency HRV; and ΔVLF, very low frequency of HRV. HRV, heart rate variability.

The results of correlation between the cardiovascular indices and possible confounding factors are shown in Table 2 and Table 3. As shown in Table 2, there were not any significant correlations between the cardiovascular indices and the impression score of ‘like-dislike’. On the other hand, a significant positive correlation between room air temperature and ΔSV (r = 0.179, *P* <0.05) was observed. There was also a tendency towards a correlation between ΔLF and room air temperature (r = 0.159, *P* <0.1). Room air temperature was significantly correlated with thermal sensation (r = 0.218, *P* <0.05).

**Table 2:** Results of correlation analysis: impression scores of ‘like-dislike’

**Indices**	**Intra-individual correlation coefficients(r)**	***P*** **value for null hypothesis is that common coefficient** ***a*** = **0**	**Significance**
ΔHR	+0.054	0.560	
ΔSV	-0.090	0.328	
ΔCO	+0.137	0.133	
ΔSBP	-0.115	0.211	
ΔDBP	-0.105	0.253	
ΔMBP	-0.131	0.153	
ΔTPR	-0.027	0.767	
ΔHF	-0.037	0.689	
ΔLF	-0.079	0.392	
ΔVLF	-0.069	0.451	
Thermal sensation	-0.067	0.465	

Correlation coefficients between each index and ‘like-dislike’ impression scores. Positive correlations mean the more pleasant (like) the impressions, the greater the indices. No significant correlations were observed. Changes from baseline: ΔHR, heart rate; ΔSV, stroke volume; ΔCO, cardiac output; ΔSBP, systolic blood pressure; ΔDBP, diastolic blood pressure; ΔMBP, mean blood pressure; ΔTPR, total peripheral resistance; ΔHF, high frequency HRV; ΔLF, low frequency HRV; and ΔVLF, very low frequency of HRV. HRV, heart rate variability.

**Table 3:** Results of correlation analysis: room air temperature

**Indices**	**Intra-individual correlation coefficients(r)**	***P*** **value for null hypothesis is that common coefficient** ***a*** = **0**	**Significance**
ΔHR	-0.149	0.103	
ΔSV	+0.179	0.049	^**^
ΔCO	+0.131	0.152	
ΔSBP	+0.046	0.614	
ΔDBP	+0.016	0.860	
ΔMBP	+0.032	0.725	
ΔTPR	-0.052	0.571	
ΔHF	+0.089	0.331	
ΔLF	+0.159	0.082	^*^
ΔVLF	-0.082	0.373	
Thermal sensation	+0.218	0.016	^**^

Correlation coefficients between each index and room air temperature. Positive correlations mean the warmer the temperature, the greater the indices. ^*^P <0.1; ^**^P <0.05. Changes from baseline: ΔHR, heart rate; ΔSV, stroke volume; ΔCO, cardiac output; ΔSBP, systolic blood pressure; ΔDBP, diastolic blood pressure; ΔMBP, mean blood pressure; ΔTPR, total peripheral resistance; ΔHF, high frequency HRV; ΔLF, low frequency HRV; and ΔVLF, very low frequency of HRV. HRV, heart rate variability.

## Discussion

The results show that several cardiovascular indices significantly correlated with the impression score. However, do they resemble the physiological response to actual hot or cold environments? Significant correlations were observed in ΔHR (positive), ΔCO (positive), ΔTPR (negative), and ΔVLF (positive). The correlation of ΔDBP (negative) and ΔMBP (negative) also tended towards significance. When we compare each index with that of the actual thermal environment, we find that, in general, when the environment is hot, cardiac output increases, total peripheral resistance decreases, heart rate increases, and blood pressure decreases, while in cold environments the reverse is true [2-4]. Therefore, these five responses (ΔHR, ΔCO, ΔTPR, ΔDBP, and ΔDBP) are similar to those found in actual thermal environments.

On the other hand, the result of ΔVLF is not so simple. The very low frequency component of HRV is known to increase in both hot and cold environments, but the increase in cold environments is greater [13]. In other words, it shows a mirrored J-shaped response. Therefore, the positive correlation between ΔVLF and the impression score does not simply correspond to the response seen in the actual thermal environment. However, if we regard this result as the right half of the mirrored J shape, we can conclude that ΔVLF is similar to the response in the actual environment. However, since we cannot identify which area of the mirrored J shape appears in this experiment, the above interpretation can be misleading. In addition, a very low frequency of HRV is, in fact, very slow fluctuation. Therefore, it is possible that heart rate fluctuations correspond to the variation among the videos. If so, ΔVLF should not be considered as either a spontaneous oscillation of heart rate or as the index of renin-angiotensin axis-related vasomotor activity [14]. We cannot conclude whether there are similarities to the actual environment based on ΔVLF.

From the viewpoint of thermoregulation, cardiac output and total peripheral resistance are the important indices because overall thermal energy flow between the body and the environment is essentially determined by these two parameters. In our results, among the other cardiovascular indices, ΔCO and ΔTPR did resemble the actual thermoregulatory responses according to the two highest correlation coefficients. This means that the responses in this study may actually contribute to the thermal energy flow to a certain degree (unfortunately, we cannot quantify this from the present data). There are two possible ways to increase cardiac output. One is to increase heart rate and the other is to increase stroke volume. In our results, ΔHR correlated significantly with the impressions, while ΔSV did not. This implies that the change in ΔCO depends on the change in ΔHR rather than on ΔSV. Notably, the change in cardiac output depends on the heart rate in actual thermoregulatory responses as well [2-4].

Regarding the relationship among cardiac output, total peripheral resistance, and blood pressure, in thermoregulatory responses the change in total peripheral resistance tends to be greater than that of cardiac output, and blood pressure decreases in hot environments and increases in cold environments [2-4]. This tendency was observed in the present study (that is, the hotter the impression, the lower the blood pressure).

As mentioned above, six cardiovascular indices showed a significant or nearly significant correlation to the impression score, and at least five of these six were in concordance with the actual thermal environment. Such hemodynamic relationships may be evidence that regional sympathetic nerve activity [15], which is specific in thermoregulatory responses, was activated in this experiment. Therefore, we can say that apparent cardiovascular responses are similar to those corresponding to actual thermal environments.

On the other hand, the effect sizes observed in this experiment are not large and there are some possible confounding factors as mentioned in the Methods. It is well known that pleasant or unpleasant feelings can induce cardiovascular responses [16]. However there were not any significant correlations with ‘like-dislike’ impression scores, indicating that pleasantness or unpleasantness did not affect this experiment. There were significant or nearly significant correlations between room air temperature and ΔSV and ΔLF, but these two indices did not correlate with the ‘hot-cold’ impression score. While room air temperature significantly correlated with thermal sensation, the effect size is much smaller than that of the ‘hot-cold’ impression score. Therefore, even considering such confounding factors, thermoregulatory-like cardiovascular responses observed in this experiment seem to be attributed to mainly ‘hot-cold’ visual information, but not conclusive.

Although this result is quite interesting, is such a response possible from the viewpoint of physiological mechanisms? One possible explanation of this phenomenon is classical conditioning [17] (so-called Pavlovian conditioning). In a study conducted by Kojo [18], participants immersed a hand in hot or cold water while hearing the sound of streaming water. Afterwards, when they heard the sound of streaming water, their skin temperature changed. This can be interpreted as a typical example that classical conditioning can also affect human temperature regulation.

In our study, however, all participants lived in Japan and most of them had never seen the Arctic Ocean, erupting volcanoes, tropical forests, or large deserts in person. Therefore, they had ‘known’ but had not ‘experienced’ the thermal conditions in the videos before the experiment. This may seem somewhat puzzling, but past studies on classical conditioning and/or brain anatomy may be able to explain this paradox.

Firstly, in classic human conditioning responses, it is known that imagery of unconditioned stimuli is more important than conditional stimuli *per se*[19]. Therefore, if mental imagery of hot or cold thermal environments were evoked by the video films, such unconditioned responses (thermoregulatory-like cardiovascular responses) could occur. Secondly, a recent brain imaging study revealed that both remembering and knowing share some common brain areas [20]. Taking these facts together, we believe that it is possible for visual information to induce thermoregulatory-like physiological responses. The question is whether these thermoregulatory-like cardiovascular responses ‘intend’ to regulate body temperature. A connectivity study indicated that the preoptic area of the hypothalamus, which is the central component of body temperature regulation, has extrinsic connections from the cerebral cortices [21]. If this pathway were involved in these responses, we could say that it is a thermoregulatory physiological response, but we cannot draw this conclusion from the present study alone.

A major weakness of this study is that we did not measure the heat radiation (mean skin temperature), heat production (O_2_ uptake), core body temperature (rectal temperature), and so on. Additional measurement cables induced great ham noises particularly on the impedance cardiograph because of the grounding condition of the laboratory. In addition, we did not want the participants to notice that we focused on the thermoregulatory responses. Small effect sizes (correlation coefficients are not large) should also be considered regardless of statistical significance. Measuring heat radiation, heat production, and core body temperature directly will uncover whether video images affect actual thermoregulation and would be an interesting future study, but more elaborate experimental design is needed. In addition this experiment was conducted in autumn in Japan for Japanese participants. It is known that thermoregulatory responses are different among seasons [22] and among ethnic groups [23]. Investigating such variations is also important from the viewpoint of physiological anthropology. These themes will be important for future studies too.

The main purpose of the human body temperature regulation system is to keep the core body temperature constant. Consequently, it is not useful for it to have sensitivity to anything that does not convey thermal energy (for example, the content of videos). Our results suggest that the human thermoregulatory system can ‘wrongly’ respond to such visual information, at least superficially. From the viewpoint of evolution, such physiological mechanisms were not maladaptive to human ancestors, as they rarely encountered situations in which visual information did not match actual thermal stimuli. However, today’s rapid increase in media technology and artificial environments may uncover this inherent defect of the thermoregulatory system, and may have negative effects on human homeostasis. To confirm such possibilities, further study is needed.

## Conclusions

We found that when participants viewed videos evoking hot or cold impressions in a thermally constant environment, their cardiovascular indices changed as if the actual thermal environment had varied corresponding to the impressions. While it is not clear how such responses were evoked, it is possible that such a propensity could result in the disturbance of homeostasis when the thermal environment and visual information are dissonant. Considering the rapid increase in media technology, it is quite easy to create dissonant artificial environments in today’s world, thus great care should be paid when designing such environments, and more study is needed.

## Abbreviations

ECG: Electrocardiography; HRV: Heart rate variability.

## Competing interests

The authors declare that they have no financial competing interests on disclosing this article.

## Authors’ contributions

JT conducted the experiment, analyzed the results, and wrote the manuscript. TN participated in the discussion and preparation of the manuscript. SW planned the experiment, participated in the discussion, and authorized the total study. All authors read and approved the final manuscript.

## Supplementary Material

Additional file 1 The explanation that the multiple regression analysis can discriminate whether the tendency is common among participants or not. Click here for file

Additional file 2: Table S2 Results of multiple regression analysis using dummy variables. Click here for file
