# Supplementary material for: Visual information without thermal energy may induce thermoregulatory-like cardiovascular responses
Source: J Physiol Anthropol. 2013 Dec 28;32(1):26. doi: 10.1186/1880-6805-32-26 (PMC3904752; doi:10.1186/1880-6805-32-26)
Supplement: Additional file 1 — The explanation that the multiple regression analysis can discriminate whether the tendency is common among participants or not. [file 1880-6805-32-26-S1.pdf]

Appendix 2

There are two sample data. In the data1 (Figure A1) independent variable and dependent variable correlates intra-individually and the tendency is common. On the other hand, in the data2 (Figure A2), independent variable and dependent variable correlates intra-individually but tendency is not common among participants. We applied multiple regression analysis for these two data. For data1, estimated common coefficient a is -0.937, and p-value for the null hypothesis $a = 0$ is 1.5×10^{-5}. For data2, estimated common coefficient a is -0.04429, and p-value for the null hypothesis $a = 0$ is 0.871. These example show that if the common coefficient a is non-zero, it means that the dependent variable y and the independent variable x are correlated intra-individually and that tendency is common among participants as described in the main manuscript.

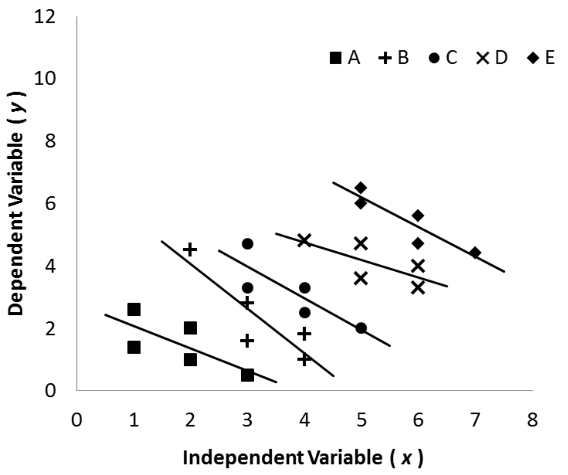

Figure A1: In case tendency is common among participants

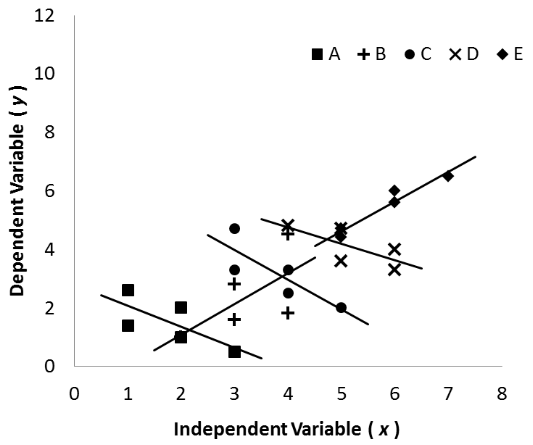

Figure A2: In case tendency is not common among participants
