# Supplementary material for: Visual information without thermal energy may induce thermoregulatory-like cardiovascular responses
Source: J Physiol Anthropol. 2013 Dec 28;32(1):26. doi: 10.1186/1880-6805-32-26 (PMC3904752; doi:10.1186/1880-6805-32-26)
Supplement: Additional file 2: Table S2 — Results of multiple regression analysis using dummy variables. [file 1880-6805-32-26-S2.pdf]

Appendix 1

Table A: Results of multiple regression analysis using dummy variables.

Dependent variable	Adjusted R^2	F-statistic	P-value of multiple regression analysis	Significance
ΔHR	0.1173	2.188	0.0102	*
ΔSV	0.05171	1.487	0.1206	
ΔCO	0.1163	2.175	0.0107	*
ΔSBP	0.1112	2.118	0.0133	*
ΔDBP	0.1238	2.263	0.0087	**
ΔMBP	0.1226	2.248	0.0081	**
ΔTPR	0.1773	2.926	0.0006	***
ΔHF	0.08626	1.843	0.0362	*
ΔLF	0.01123	1.101	0.3625	
ΔVLF	0.07407	1.715	0.0567	†
TS	0.2393	3.810	1.683e-05	***

Please note that the significance of multiple regression analysis does NOT mean the impression score of "hot-cold" and dependent variables correlate significantly. Degree of freedom is 119 for each row, and F-statistic is on 15 and 119.
